# Supplementary material for: A Phase Ib Expansion Cohort Evaluating Aurora A Kinase Inhibitor Alisertib and Dual TORC1/2 Inhibitor Sapanisertib in Patients with Advanced Solid Tumors
Source: Cancers (Basel). 2024 Apr 10;16(8):1456. doi: 10.3390/cancers16081456 (PMC11048245; doi:10.3390/cancers16081456)

Supplementary Table S1: Patient outcomes by tumor type across all cohorts

Tumor Type	Number of Patients	Median Weeks on Treatment (Range)	Best response by RECIST Number (Percent)				Reason for Study Discontinuation Number (Percent)				
			Partial Response	Stable Disease	Progressive Disease	Not Evaluable	Progression	Clinical Progression	Toxicity	Unrelated Medical Comorbidities	Patient Choice
Pancreatic Adenocarcinoma	14	9.2 (4-47)	0/14 (0%)	5/14 (36%)	3/14 (21%)	6/14 (43%)	7/14 (50%)	3/14 (21%)	2/14 (14%)	1/14 (7%)	1/14 (7%)
Breast Adenocarcinoma ER+/HER2-	7	8.9 (2-14.9)	1/7 (14%)	3/7 (43%)	2/7 (29%)	1/7 (14%)	3/7 (43%)	1/7 (14%)	3/7 (43%)	0/7 (0%)	0/7 (0%)
Breast Adenocarcinoma TNBC	2	5.3 (3.6-7)	0/2 (0%)	0/2 (0%)	1/2 (50%)	1/2 (50%)	1/2 (50%)	1/2 (50%)	0/2 (0%)	0/2 (0%)	0/2 (0%)
Colorectal Adenocarcinoma	4	9.4 (4.9-12.7)	0/4 (0%)	0/4 (0%)	4/4 (100%)	0/4 (0%)	4/4 (100%)	0/4 (0%)	0/4 (0%)	0/4 (0%)	0/4 (0%)
Ovarian Serous Carcinoma	2	12.1 (11.1-13)	0/2 (0%)	2/2 (100%)	0/2 (0%)	0/2 (0%)	2/2 (100%)	0/2 (0%)	0/2 (0%)	0/2 (0%)	0/2 (0%)
Renal Cell Carcinoma	1	1.1 (N/A)	0/1 (0%)	0/1 (0%)	0/1 (0%)	1/1 (100%)	0/1 (0%)	1/1 (100%)	0/1 (0%)	0/1 (0%)	0/1 (0%)
Uterine Serous Carcinoma	1	10 (N/A)	0/1 (0%)	0/1 (0%)	1/1 (100%)	0/1 (0%)	1/1 (100%)	0/1 (0%)	0/1 (0%)	0/1 (0%)	0/1 (0%)

Supplementary Table S2: Treatment-related adverse events attributed to one or both drugs occurring in at least 10% of patients across treatment groups

	**Alisertib Lead-In N=10** Patients A-1 through A-10		**Sapanisertib Lead-In N=10** Patients S-1 through S-10		**Pancreatic Cancer Expansion N=11** Patients P-1 through P-11		**All Treatment Groups N=31**		
**Number (percent)**	**Grade 1/2**	**Grade 3/4**	**Grade 1/2**	**Grade 3/4**	**Grade 1/2**	**Grade 3/4**	**Grade 1/2**	**Grade 3/4**	**Total**
Fatigue	2	1	4	0	6	0	12 (39%)	1 (3%)	13 (42%)
Mucositis	3	2	2	0	1	1	6 (19%)	3 (10%)	9 (29%)
Hyperglycemia	2	1	2	0	3	0	7 (23%)	1 (3%)	8 (26%)
Nausea	2	0	1	0	4	0	7 (23%)	0	7 (23%)
Neutropenia	0	1	1	0	0	3	1 (3%)	4 (13%)	5 (16%)
Thrombocytopenia	2	0	1	0	1	1	4 (13%)	1 (3%)	5 (16%)
Diarrhea	1	0	1	0	2	0	4 (13%)	0	4 (13%)
Cognitive disturbance	0	0	1	0	2	0	3 (10%)	0	3 (10%)
Vomiting	1	0	0	0	2	0	3 (10%)	0	3 (10%)
Anorexia	1	0	0	0	2	0	3 (10%)	0	3 (10%)
Alopecia	3	0	0	0	0	0	3 (10%)	0	3 (10%)

Supplementary Figure S1

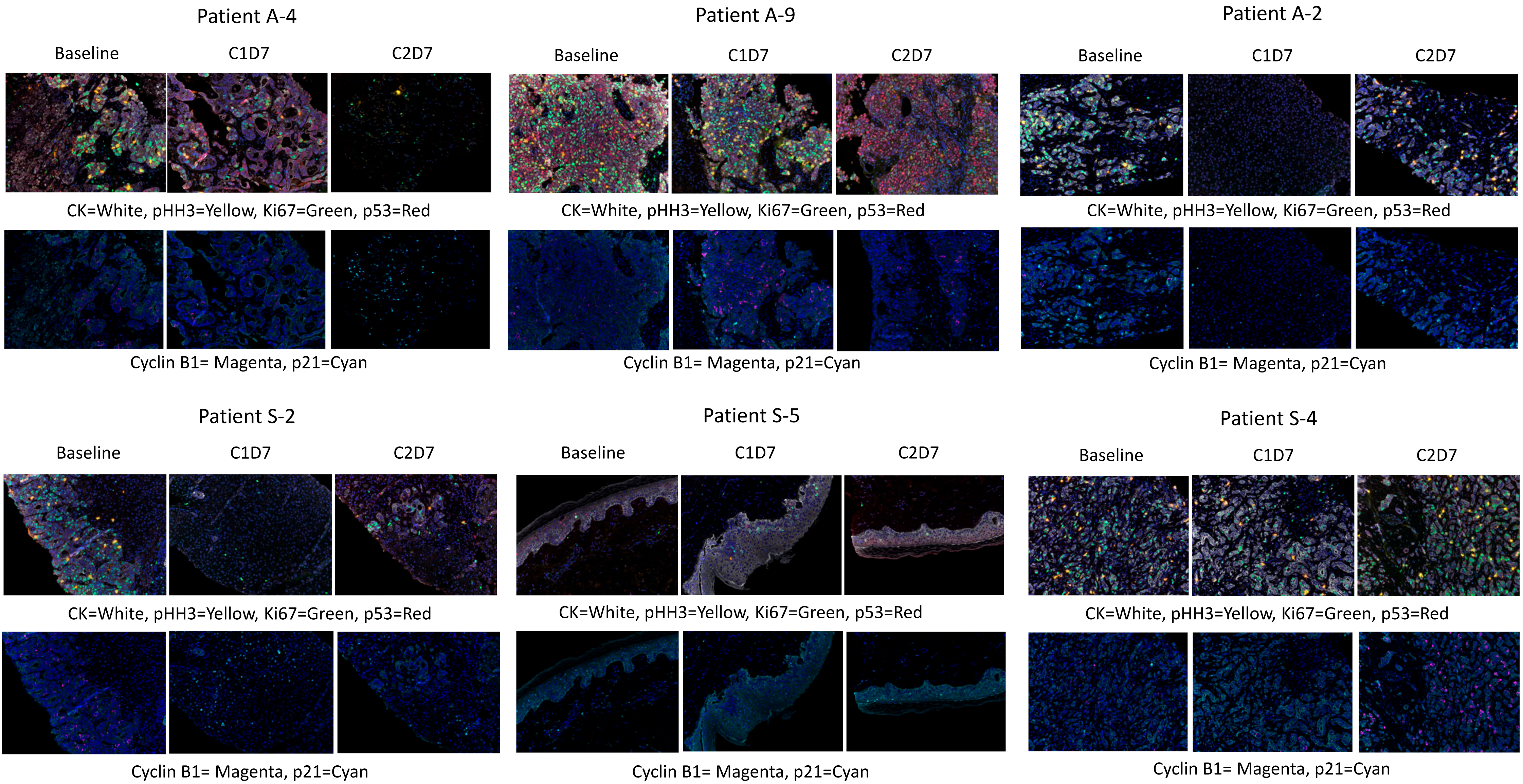

Supplementary Figure S2

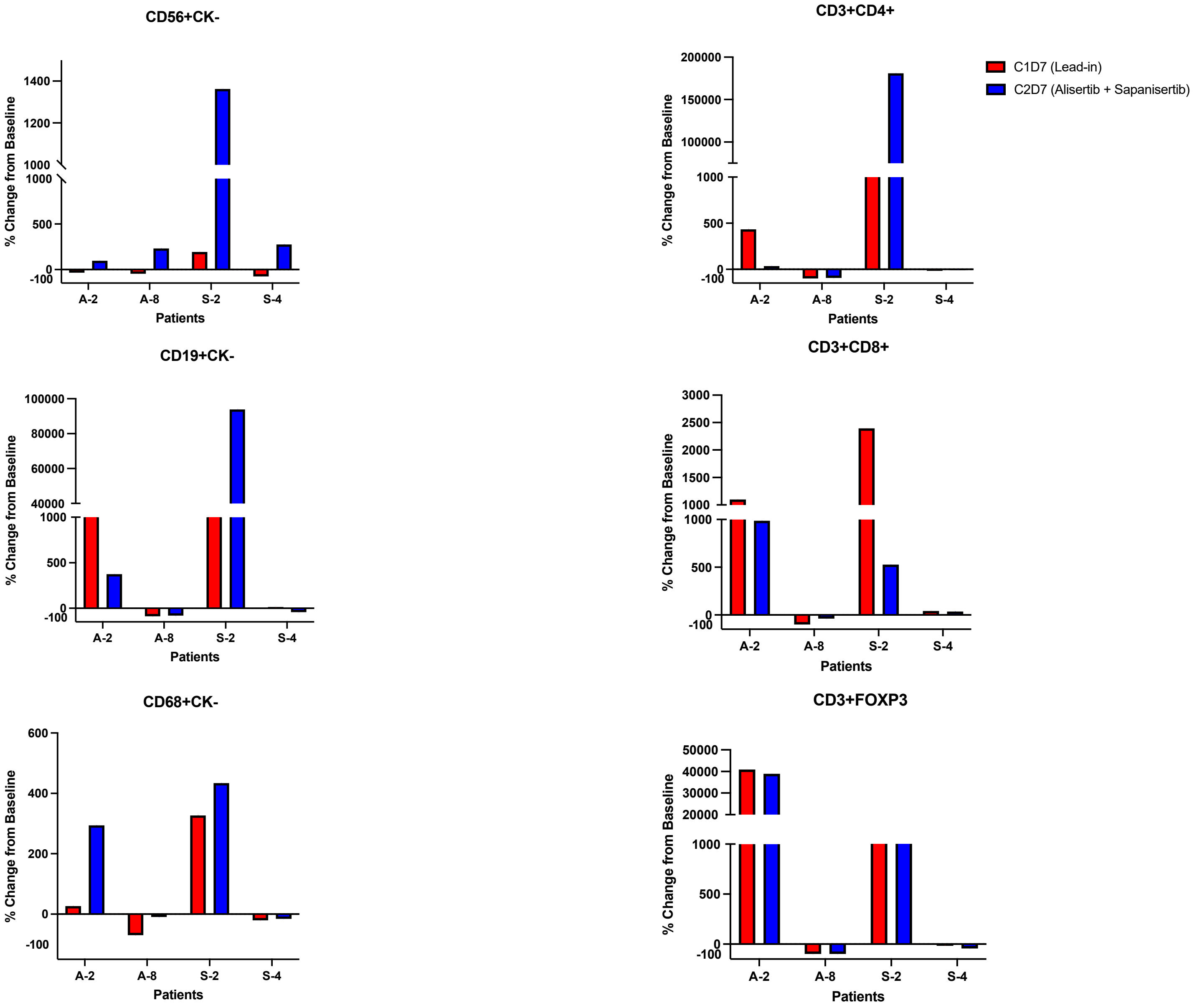

Supplement: Supplementary file 1 [file cancers-16-01456-s001.zip › cancers-2889405-supplementary.pdf]
